# Diel activity structures the occurrence of a mammal community in a human‐dominated landscape

**DOI:** 10.1002/ece3.10684

**Published:** 2023-11-02

**Authors:** Amy E. Mayer, Laken S. Ganoe, Charles Brown, Brian D. Gerber

**Affiliations:** ^1^ Department of Natural Resources Science University of Rhode Island Kingston Rhode Island USA; ^2^ Rhode Island Department of Environmental Management Division of Fish and Wildlife West Kingston Rhode Island USA

**Keywords:** community, diel, habitat, mesocarnivore, occupancy camera traps, occupancy

## Abstract

Anthropogenic developments alter the environment and resources available to wildlife communities. In response to these real or perceived threats from this development, species may adjust their spatial occurrence. Additionally, wildlife species may adjust when in diel time (24‐h light–dark cycle) they occupy sites on the landscape to adapt to changing conditions. However, many wildlife studies only focus on where a species does and does not occur, ignoring how species may shift their diel activity at sites to mitigate threats. We used a multi‐state diel occupancy modeling framework to investigate how a community of mammals (mesocarnivores, urban‐adapted omnivores, and herbivore/small mammals) respond to differing levels of anthropogenic development and forest cover across two climatic seasons. We collected camera trap data at 240 survey locations across the summer and winter of 2021–2022. We modeled multi‐state diel occupancy for 14 mammal species with extent of development/forest and season hypothesized to influence diel occupancy and season hypothesized to influence the probability of detection. We found that all species displayed heterogeneity in both diel occupancy and detection either by extent of development/forest and or season. Within the mesocarnivore species group, coyote and red fox were less sensitive to development and had higher occupancy probability at these sites in general but used them more during the night, while more sensitive mesocarnivores including fisher and bobcat occupied the day state only when there was increasing forest cover. Our results highlight the importance of incorporating diel activity in habitat modeling to better understand the relationship between a species and its landscape, particularly in a region that is vulnerable to increased anthropogenic pressure.

## Graphical abstract

Anthropogenic developments alter the environment and resources available to wildlife, and in response wildlife may alter both where and when they occur on the landscape. We used a multi‐state diel occupancy model to investigate how a community of mammals respond to differing levels of development and forest cover across seasons. We found that many mammals vary in both spatial and diel occupancy in response to anthropogenic development.

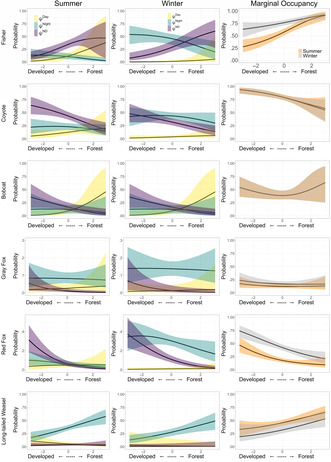

## 1 INTRODUCTION

Understanding the relationship between a species and the physical environment where it occurs and how that relationship is impacted by landscape change and anthropogenic pressures is a fundamental aim in wildlife ecology (Mitchell, 2005). This relationship is typically studied by examining the spatial resources, or habitat, within an animal's home range (Aarts et al., 2008; Johnson, 1980; Manly et al., 2002). In recent years, the definition of what is considered a species' habitat has evolved to incorporate a temporal component at varying scales to also describe when a species is likely to use or select for a particular landscape or resource (Kearney, 2006). Understanding the variation in both temporal and spatial habitat use allows for more accurate definitions of a species' habitat (Morano et al., 2019) and thus improves the overall understanding of how a species interacts with their environment (Enright, 1970; Farris et al., 2020).

The consideration of how animals vary their temporal use of a landscape is becoming a more frequent consideration in describing an animals' habitat (Wolkovich et al., 2014). Studies have shown seasonally driven variation in spatial landscape use in response to the availability of resources (Svoboda et al., 2019), abiotic conditions such as temperature (Street et al., 2015; Thompson & Colgan, 1994), and due to life history traits such as breeding and migration patterns (Brinkman et al., 2005; Phillips et al., 1998; Plowman et al., 2006; Sabine et al., 2002). While seasonal variation is an important consideration in understanding species' spatial landscape use, studies have also shown that landscape use can vary temporally on a finer 24‐h, or diel scale (Gallo et al., 2022; Gaston, 2019; Gaynor et al., 2018; Morano et al., 2019). For example, daytime predation risk may lead to more night activity in prey species (Swarts et al., 2009), and alternatively, increased nighttime predation pressure can drive prey to become more diurnal (Monterroso et al., 2013). In areas where animals must coexist with humans, a species' temporal activity can be impacted by human‐driven landscape change (Frey et al., 2020), competition, or predation from introduced/non‐native species (Gerber et al., 2012), and the real or perceived risk from human activity on the landscape (Cox et al., 2023; Wang et al., 2015).

Individual species or communities may react differently to the cost or benefit of using a space during a given diel time period or season (Ellington et al., 2020; Gaynor et al., 2018; Murray & St. Clair, 2015; Riley et al., 2003). For example, studies have shown that predators such as pumas (*Puma concolor*) and bobcats (*Lynx rufus*) are more active at night in areas of higher human disturbance (Lewis et al., 2015), and white‐tailed deer (*Odocoileus virginianus*) and American black bear (*Ursus americanus*) can become more nocturnal during certain times of the year in response to hunting activity (Hubbard et al., 2022; Kilgo et al., 1998). Coyotes (*Canis latrans*) are often more nocturnal in areas where the population has been historically exploited (Atwood et al., 2004) and their diel activity can shift based on the extent and temporal patterns of human disturbance (Melville et al., 2020; Way et al., 2004). As such, wildlife can experience pressure not only from spatial habitat loss, but temporal habitat loss as well. While the loss of spatial habitat is generally considered more impactful to a species, the loss of access to a space at certain times of the day may be equivalent when it leads to the loss of important temporal resources (e.g., prey availability). As such, a species' ability to adapt to both spatial and temporal pressures can be an important factor in their ability to persist within a changing landscape.

To make inference on a species' spatial and temporal landscape use requires consideration of both dimensions simultaneously. Commonly in mammal research, occupancy models are used to make inference on the spatial occurrence of a species while accommodating for variation in detection probability (MacKenzie et al., 2017). However, inference on occurrence is typically made without regard to variation in diel activity across sites. Recently, Rivera et al. (2022) demonstrated the utility of incorporating diel time as an explicit state in an occupancy model, via the MSDOM. By doing so, inference can be made at how site occurrence varies across different periods of times, such as day only, night only, and both day and night. Further, detection probability can be made to vary by these states, which accommodates a source of heterogeneity that is commonly not considered; namely, that many mammal species are active across different time periods at different rates depending on the environmental context (Gallo et al., 2022).

Motion‐triggered camera traps have been a commonly used tool in wildlife research for many years (Kucera & Barrett, 2011). Camera traps can passively collect continuous time‐stamped data on species occurring on the study landscape. The relatively low cost along with developments and improvements in camera design means that researchers are able to easily and affordably cover wide survey areas over multiple seasons. As detections of multiple species are collected continuously over time, this data can be used to examine interactions between species, such as spatial and temporal partitioning (Frey et al., 2017; Rota et al., 2016). We can also compare how different species within a study area or community react to the same anthropogenic pressures both spatially (e.g., selecting for forest over development) and temporally (e.g., selecting undisturbed areas during the day but developed areas at night). By making inference to an entire wildlife community, we gain a better understanding of not only critical areas for community‐scale conservation, but also the critical diel periods in which the community of species use these areas.

In this study, we aim to understand a community of mammals in three functional guilds – mesocarnivores, urban adapted omnivores, and herbivore/small mammals – and how they adjust their habitat in space and diel time in response to changing seasons, and varying levels of natural forest and anthropogenically altered landscapes. We do so by fitting the MSDOM to make inference on the drivers of when and where species occur and evaluate the consistency of the factors affecting their occurrence. We hypothesized that occupancy for all species would vary in diel time both seasonally (winter vs. summer) and by the amount of forest cover and anthropogenic disturbance surrounding a survey location. Forests are an important landcover that provides cover and food sources for many species in our study area, as such we predicted that increasing amounts of forest cover would have a positive effect on occurrence in the daytime for mesocarnivores and herbivores/small mammals. Alternatively, anthropogenic factors including development and roads may be perceived as areas of high risk and thus avoided during the day when human interference is greatest and only used at times when disturbance is minimal (e.g., nighttime; Wang et al., 2015). Thus, we hypothesized that mesocarnivores and urban‐adapted omnivores would primarily use these locations at night. Additionally, we hypothesized that carnivores would have higher occupancy temporally and spatially when and where higher numbers of available prey species were detected. We hypothesized that herbivores/small mammals would benefit from a lack of larger predator activity at developed sites during the day and have higher occupancy in daytime at more developed sites. Overall, we expected that the extent of anthropogenic disturbance would have greater negative effect on occupancy during the day for mesocarnivore species, while the extent of human disturbance would have less of an effect on occupancy in this state for urban‐adapted omnivores, herbivores, and small mammals. Due to differences in the amount of available day and night hours between the survey seasons, we hypothesized that diel occupancy would vary seasonally with winter positively affecting occupancy at night for all species, while mesocarnivores and urban adapted omnivores would have a higher probability of occupying sites in the day during the summer only.

## 2 METHODS AND MATERIALS

**FIGURE 1:**
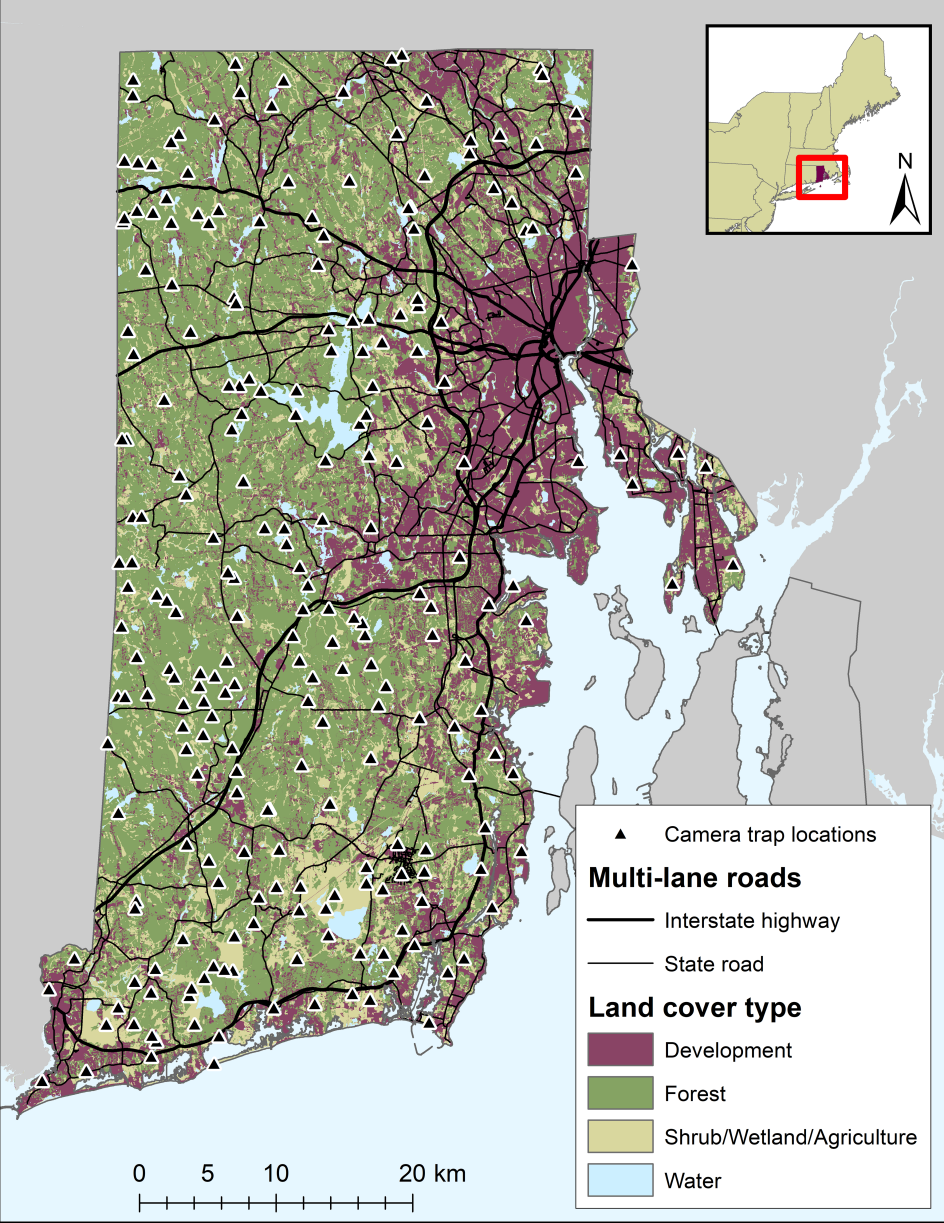
The study area map of Rhode Island in the northeast United States. The state is highly forested despite being the second most densely populated state in the U.S. Camera traps were located throughout the mainland on parcels of conservation land.

We sampled the terrestrial mammal community in Washington, Kent, Providence and Bristol counties on the mainland of Rhode Island in the Northeastern United States (Figure 1). The natural landscape of Rhode Island is primarily forested (55.6% of total land area), however it is also the second most densely populated state in the United States (U.S. Census Bureau, 2021). The characteristics of the state, with both a high percentage of forest and a high percentage of anthropogenic pressure, make it an ideal location to understand how species respond and adapt spatially and temporally to changes on the natural landscape. Our analysis focused on 14 mammal species that we characterized in three groups based on diet, ecological niche, and expected reaction to increased human development: mesocarnivores, urban‐adapted omnivores, and herbivore/small mammals. The mesocarnivore species group consisted of predators in the study area and included bobcat (*Lynx rufus*), fisher (*Pekania pennanti*), coyote (*Canis latrans*), red fox (*Vulpes vulpes*), gray fox (*Urocyon cinereoargenteus*), and long‐tailed weasel (*Mustela frenata*); the urban‐adapted omnivore species consisted of species that commonly occur in suburban environments and included raccoon (*Procyon lotor*), Virginia opossum (*Didelphis virginiana*), and striped skunk *(Mephitis mephitis*); and the herbivore/small mammal group consisted of common prey species and included: white‐tailed deer (*Odocoileus virginiana*), eastern cottontail (*Sylvilagus floridanus*), gray squirrel (*Sciurus carolinensis*), red squirrel (*Tamiasciurus hudsonicus*) and eastern chipmunk (*Tamias striatus*). Note that the coyote could also be grouped as an urban‐adapted omnivore, however, we group them as a carnivore because they are still highly carnivorous in urban environments (Larson et al., 2020) and may fill the role of top predator in an ecosystem where apex predators are absent (Crooks & Soulé, 1999; Jones et al., 2016). Additionally, people's perception of the species aligns more closely with the other mesocarnivores compared with more commonly encountered species such as raccoons, striped skunks, and Virginia opossums.

We collected continuous camera trap data in 6‐week increments across two seasons in 2021 and 2022: Summer (May 26 to September 16, 2021) and winter (October 28, 2021 to March 18, 2022). We placed two north‐facing camera traps (Browning Strike Force HD Pro XD, Browning, Morgan, UT, USA or Bushnell TrophyCam, Bushnell Outdoor Products, Overland Park, KS, USA) within 100 m of one another at selected survey sites (*n* = 240 sites). Each camera in a survey site was placed in a different micro‐habitat and 50 cm–1 m off the ground to maximize detections of mammal species within the site (Mayer et al., 2022). The average spacing between survey sites of 1.79 km (*SD* = 1.05; Figure 1). Cameras were set to take a burst of three photos when triggered by movement with a 10 s delay in between bursts. We applied scent lure (Caven's Gusto, Minnesota Trapline Products, Pennock, MN, USA) 3–4 m in front of each camera, approximately 1–2 m off the ground once at the initial setting of the camera traps.

We used the photo data management program Camelot (Hendry & Mann, 2018) to catalog and identify all photos collected during each survey period. The detections collected from both cameras at a site were merged and were treated as one site. Detections of the same species at the same site were considered independent if there was more than one individual in the frame, or the detections occurred >20 min from a previous detection. We used the R package suncalc (Thieurmel & Elmarhraoui, 2022) to categorize each independent detection as either “day” (captured after sunrise and before sunset) or “night” (captured after sunset and before sunrise). We created a detection matrix with a 24‐h occasion time for each species during both the winter and summer survey periods. We determined the appropriate occasion start time for each species by examining the radial activity density distribution plots (Niedballa et al., 2016) of all independent detections and selected the hour with the fewest detections during each season (Appendix 1). To construct the detection matrix for the MSDOM, we coded each occasion in the matrix as either state 1 (no detection), state 2 (daytime detection), state 3 (nighttime detection), or state 4 (both day and night detections in a single occasion).

We quantified the amount of forest and anthropogenic disturbance by summarizing four landscape variables within a 1 km buffer zone of each survey site to capture the variation between each survey site as well as the broader spatial variation around a survey site that can influence a species' occurrence at that site. We used the Rhode Island Land Use and Land Cover 2020 dataset (https://www.rigis.org/datasets/edc::land‐use‐and‐land‐cover‐2020/, accessed July 31, 2023) to calculate the proportion of each buffer area that contains forests (*forest.cover*) and residential development (*residential*), and used the Rhode Island DOT data set (https://www.rigis.org/datasets/edc::ridot‐roads‐2016/, accessed July 31, 2023) to calculate the road density within the buffer (km roads per km^2^; *road.density*). We created a housing unit index for each buffer by using the 2020 U.S. Census Data (https://www.census.gov/geographies/reference‐files/2020/geo/2020addcountlisting.html, accessed July 31, 2023). We standardized the housing units per census block into units per square kilometer and multiplied this by the area of each block that is within the buffer area to get a single housing unit density for each survey site buffer (*housing*). This index was used as an approximation of the number of people living within the buffer zone of each survey site. Separate inference from each of these variables was determined to not be possible given the high‐level of pair‐wise correlation among them (|*r*| > .68). As such, we applied a principal component analysis (Jollife & Cadima, 2016) to the *forest.cover*, *residential*, *housing*, and *road.density* variables to get a single metric (first principle component, explaining 85.1% variation) where negative values indicate more anthropogenic features and positive values indicate more available forest (hereafter, *PCA*; Appendix 2). We also created an available prey index for each site based off the number of independent detections of certain small mammal species that are common prey of all mesocarnivore species in our analysis. The prey species included: flying squirrel (*Glaucomys volans*), gray squirrel (*Sciurus carolinensis*), eastern cottontail (*Sylvilagus floridanus*), eastern chipmunk (*Tamias striatus*), red squirrel (*Tamiasciurus hudsonicus*), and other small rodents (i.e., mice, rats, voles, moles). We recognize this is a crude index that aims to capture spatial variation in potential prey availability and does not capture the range of all food components of each species' diet. However, it has the potential to capture an otherwise missed source of spatial variation. For each survey site we combined all prey species detections and calculated the average number of detections overall per trap during night and day, separately, for both the summer and winter seasons (*prey*). Lastly, by stacking the detection matrices for each survey period by species, we created a seasonal covariate (*season*) as a categorical variable where summer was the reference category.

We defined the MSDOM in terms of four mutually exclusive states: (1) no use, (2) day use, (3) night use, and (4) night and day use, where we are interested in estimating the respective state occupancy probabilities 𝝍^1^, 𝝍^2^, 𝝍^3^, and 𝝍^4^. We considered both the “full” and “reduced” model parameterizations outlined by Rivera et al., 2022, where the full models allow 𝝍^4^ to be estimated independently from 𝝍^2^ and 𝝍^3^, and reduced models are where 𝝍^4^ is derived from the combination of those parameters (𝝍^4^ = 𝝍^2^ × 𝝍^3^). Therefore, the full model allows 𝝍^4^ to be higher or lower than what we would expect given independent use of day‐only and night‐only sites. We linked site covariates to model parameters using the multi‐nominal logit link function where state 1 was the reference category (see Rivera et al., 2022).

Each species' data was separately fit to a small set of models. For the urban‐adapted omnivores and herbivore/small mammal species, we considered a total of 10 competing models that incorporated combinations of variables modeling state‐occupancy: full and reduced parameterizations as (1) no covariate models, (2) single covariate models (*season* or *PCA*), (3) additive covariate models (*season* + *PCA*), and (4) interaction covariates models (*season* ✕ *PCA*). For mesocarnivore species, we included four additional models within the model set that included the prey variable using the full and reduced parameterizations as a single covariate model and additive covariate with season. For all models, we considered detection probability to vary by state and season. Due to our study area occurring in a temperate landscape dominated by deciduous vegetation, we expect that changes in both the vegetation cover and availability of resources between winter and summer would have an effect on our ability to detect most species. Further, there are differences in seasonal behaviors for many species in our study due to life history traits such as breeding and young‐rearing, and we hypothesized that these seasonal behavioral shifts would add to variation in our ability to detect a species.

All models were fit in a Bayesian framework where parameters of non‐covariate models were given relatively uninformative prior probability distributions (i.e., probability parameters were either Beta(1, 1) or Dirichlet(1, 1, 1, 1)). Otherwise, covariate models with parameter effects were given relatively diffuse prior distributions of Logistic(0, 1) (Northrup & Gerber, 2018). We fit all models in JAGS version 4.3.1 (Plummer, 2003) with the runjags package (Denwood, 2016) in R version 4.2.3 (R Core Team, 2023). We assessed convergence using the Gelman‐Rubin diagnostic (Gelman & Rubin, 1992) to ensure that all values were <1.1 and by visually examining trace plots of the posterior distributions. We compared models using the conditional predictive ordinate (CPO; Hooten & Hobbs, 2015) and evaluated evidence of an effect with the most supported model by deriving the probability of an effect being greater than zero, 𝜂 = (Pr > 0); when this probability is high (≥.90) indicates strong evidence of a positive effect, while a low probability (≤.10) indicates strong evidence of a negative effect.

## 3 RESULTS

During our total study period, we observed and collected camera trap data for 14 mammal species in three groups – mesocarnivores, urban‐adapted omnivores, and herbivore/small mammals – across both field seasons. During the summer season we observed a total of 25,137 independent detections from those species across 20,219 trap nights, and during the winter we observed 24,783 independent detections across 23,277 trap nights (Table 1).

**TABLE 1:** Total number of independent detections across all survey sites for each species across two survey seasons.

	Summer detections		Winter detections	
	Day	Night	Day	Night
Bobcat	41	27	26	84
Fisher	171	164	65	618
Coyote	297	376	162	926
Red fox	105	94	100	416
Gray fox	57	78	7	171
Long‐tailed weasel	32	448	1	134
White‐tailed deer	3326	1705	1594	269
Eastern cottontail	300	331	18	1130
Gray squirrel	8368	185	11,940	1306
Red squirrel	2299	109	2277	238
Eastern chipmunk	3166	10	870	32
Striped skunk	4	140	0	36
Virginia opossum	13	1022	1	232
Raccoon	217	2052	30	2100

*Note*: Day detections occurred after sunrise and before sunset, and night detections occurred after sunset and before sunrise.

We found the full MSDOM with state occupancy varying by *season* and *PCA* (additive combination) was the most supported model for four mesocarnivore species (coyote, fisher, gray fox, red fox), three herbivore/small mammals (white‐tailed deer, eastern cottontail, and red squirrel), and one urban‐adapted omnivore (raccoon; Appendix 3). The full model where occupancy and detection only varied by *season* was the most supported model for two herbivore/small mammals (eastern chipmunk and gray squirrel), while the full model occupancy varying by *PCA* was the most supported model for one mesocarnivore (bobcat). The reduced multi‐state model was most supported for one mesocarnivore (long‐tailed weasel) and two urban‐adapted omnivores (Virginia opossum and striped skunk); as such, there is no evidence that these species use the day/night state more than or less than expected from how they use the day and night states. Models including a covariate for amount of prey availability at a site was not the most supported model for any of the mesocarnivore species.

**FIGURE 2:**
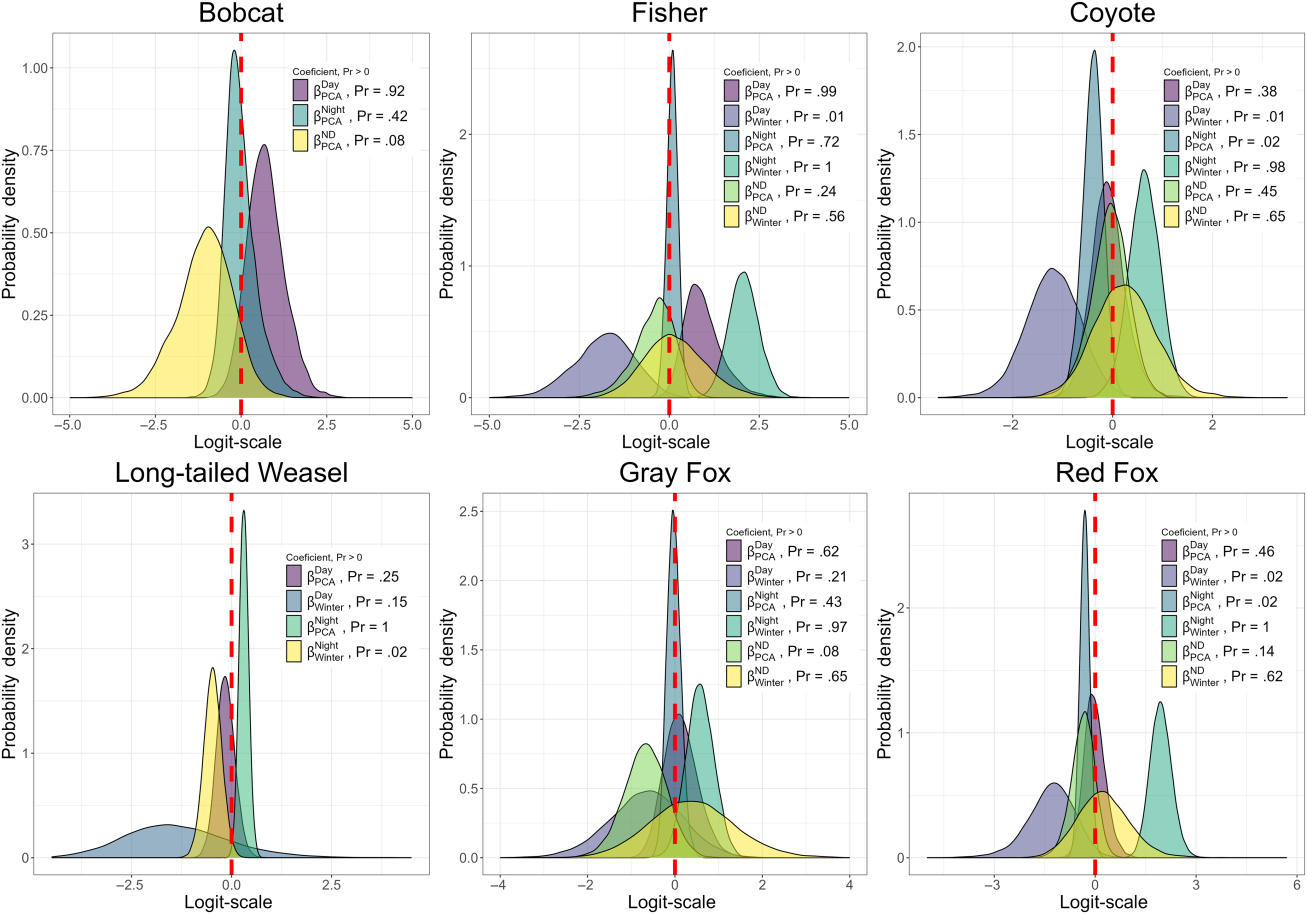
Posterior probability distributions of parameter effects or coefficients (*β*) on state‐occupancy for the most supported model for each carnivore species. A probability >.9 indicates strong evidence of positive support for the parameter, and a probability <.1 indicates strong evidence of negative support for the parameter. Results are given on the logit scale and are in relation to the probability of a site not being occupied by each species. A probability >.9 for PCA coefficients indicates support for higher probability of occupancy with increasing forest cover, while a probability <.1 for PCA coefficients indicates support for higher probability of state occupancy with increasing anthropogenic development (roads and residential development). A probability >.9 for the winter coefficient indicates higher probability of state occupancy during the winter season, and a probability <.1 indicates support for higher probability of occupancy during the summer when compared to no occupancy.

**FIGURE 3:**
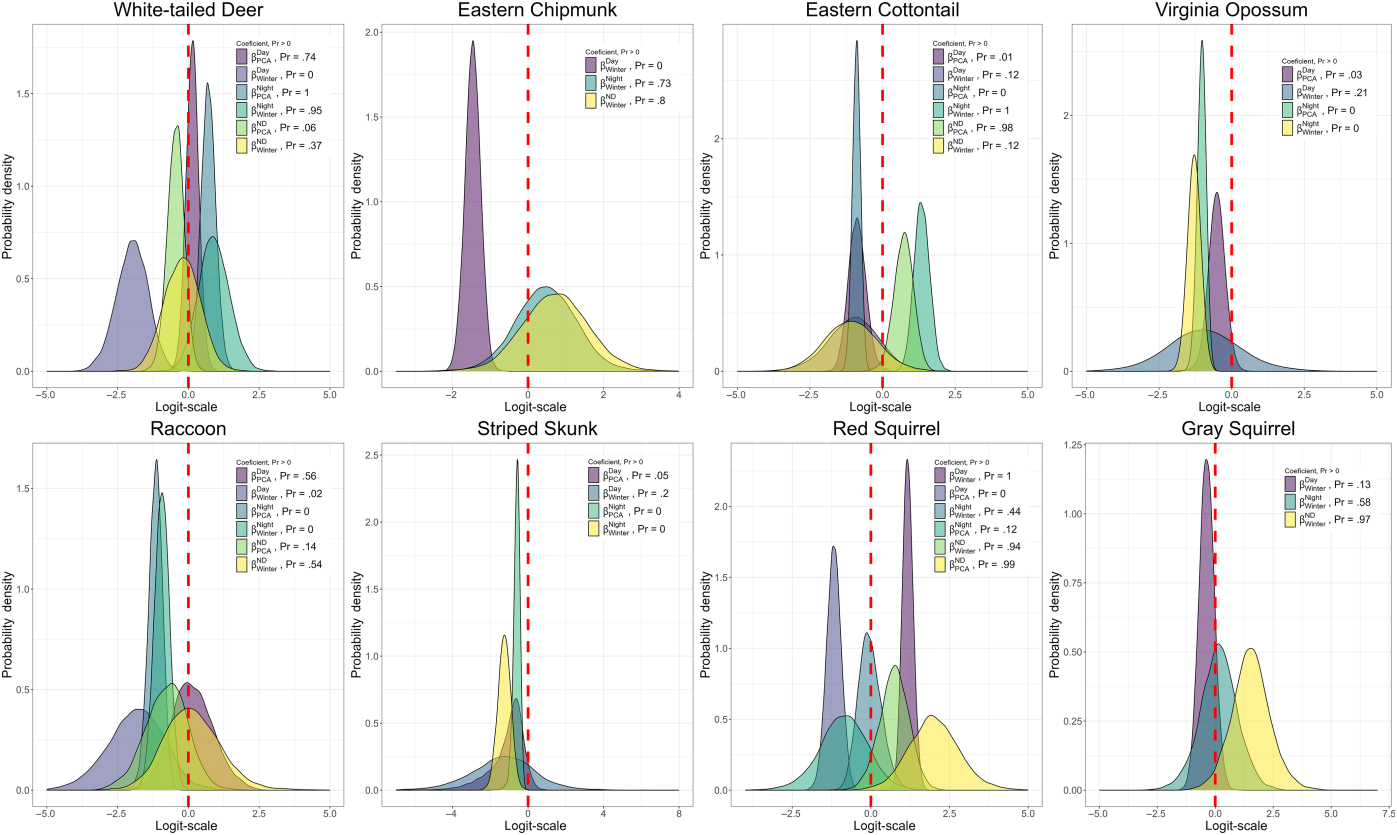
Posterior probability distributions of parameter effects or coefficients (*β*) on state‐occupancy for the most supported model for urban‐adapted omnivores and herbivore/small mammal species. A probability >.9 indicates strong evidence of positive support for the parameter, and a probability <.1 indicates strong evidence of negative support for the parameter in the model. Results are given on the logit scale and are in relation to the probability of a site not being occupied by each species. A probability >.9 for PCA coefficients indicates support for higher probability of occupancy with increasing forest cover, while a probability <.1 for PCA coefficients indicates support for higher probability of state occupancy with increasing anthropogenic development (roads and residential development). A probability >.9 for the winter coefficient indicates higher probability of state occupancy during the winter season, and a probability <.1 indicates support for higher probability of occupancy during the summer when compared to no occupancy.

For the eight species that had a seasonal parameter for state occupancy in the most supported model, species with a higher probability of occurrence in the day‐use state during summer (𝜂 < 0.1) – relative to not occupying a site – included three mesocarnivores (fisher, coyote, red fox; Figure 2), one urban‐adapted omnivore (raccoon), and two herbivore/small mammals (white‐tailed deer and eastern chipmunk; Figure 3), while red squirrel was the only species to have a higher probability of occupying a site in the day‐use state during winter (𝜂 > 0.9, Figure 3). In relation to not occupying a site, three mesocarnivores (fisher, coyote, and red fox), and two herbivore/small mammals (white‐tailed deer and eastern cottontail) have a higher probability of occupying a site in the night‐use state during the winter season, while one mesocarnivore (long‐tailed weasel), and three urban‐adapted omnivores (Virginia opossum, raccoon, and striped skunk) have a higher probability of occurrence in the night‐use state during the summer (Figures 2 and 3). Red squirrel and gray squirrel were the only species with support for seasonal difference in occupancy in day/night‐use state with a higher probability of occupancy in day/night‐use state during the winter in relation to not occupying a site (Figure 3).

We found the most supported model for all species – excluding gray squirrel and eastern chipmunk – included a parameter for *PCA*, indicating an effect of forest cover and anthropogenic development. With increasing forest cover, two mesocarnivores had a higher probability of occupying the day state (bobcat (𝜂 = 0.92) and fisher (𝜂 = 0.99)), and long‐tailed weasel (𝜂 = 0.25) and white‐tailed deer (𝜂 = 1.00) had a higher probability of occupying a site at night. Two herbivore/small mammals (eastern cottontail (𝜂 = 0.98) and red squirrel (𝜂 = 0.99)) had a higher probability of occupying the day/night state as forest cover increased at a site (Figures 2 and 3; Appendix 4). As the extent of anthropogenic features increased at a site, two urban‐adapted omnivores (striped skunk (𝜂 = 0.05) and Virginia opossum (𝜂 = 0.03)) and two herbivore/small mammals (eastern cottontail (𝜂 = 0.01), and red squirrel (𝜂 = 0.00)) had a higher probability of occupying the day‐use state, while two mesocarnivores (coyote (𝜂 = 0.02) and red fox (𝜂 = 0.02)), three urban‐adapted omnivores (raccoon (𝜂 = 0.00), striped skunk (𝜂 = 0.00) and Virginia opossum (𝜂 = 0.00)) and one herbivore/small mammal (eastern cottontail (𝜂 = 0.00)) all were more likely to occupy the night‐use state. The higher probability of striped skunks, Virigina opossums, and eastern cottontails occupying both day‐use state and the night‐use state as the extent of anthropogenic features increases is indicative of a higher probability of using these sites compared to forested sites regardless of diel time. Two mesocarnivores (bobcat (𝜂 = 0.08), gray fox (𝜂 = 0.08)) and white‐tailed deer (𝜂 = 0.06) were more likely to occupy the day/night‐use state with increased development (Figures 2 and 3; Appendix 4).

**FIGURE 4:**
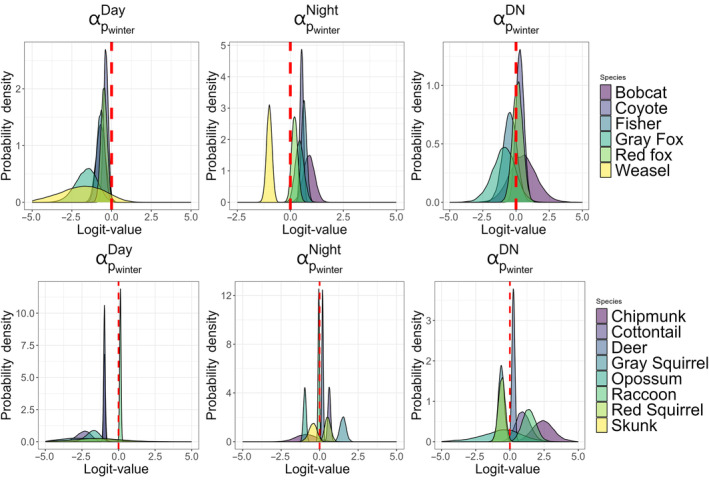
Posterior probability distributions of the coefficient (α) for the effect change in season from summer to winter on the detection probability by species and state. For species where the reduced model was most supported, there are no plots for day/night detection (α^DN^; long‐tailed weasel, Virginia opossum and striped skunk). Species with posterior distributions >0.0 on the logit scale have a higher likelihood of being detected in each state during the winter season, and those with probability densities <.0 on the logit scale have a higher likelihood of being detected in each state during the summer season.

We found all six mesocarnivore species had a higher probability of daytime detection during the summer months, and all but the long‐tailed weasel had a higher probability of detection at night during the winter (Figure 4; Appendix 5). Similarly, of the three urban‐adapted omnivores and five herbivore/small mammals, only red squirrels had a higher probability of detection during the day in winter, although the coefficient was not large (mean = 0.127, 95% CI = 0.035–0.219). Additionally, there was variation in the probability of detection by season at night for these species. Two typically diurnal species, gray squirrel, and red squirrel, had a higher probability of detection at night during the winter season, while typically nocturnal urban‐adapted omnivores, Virginia opossum, and striped skunk, had a higher probability of detection at night during the summer season (Figure 4). The seasonal effect on the probability of detection during the day/night state had more variation among the groups of species. Note that the species whose most supported model was the reduced MSDOM (long‐tailed weasel, striped skunk, and Virginia opossum) are not reported as there was no estimated effect. In the mesocarnivore group, there was moderate support for a higher probability of detection in day/night in the summer season for gray fox (𝜂 = 0.14) and fisher (𝜂 = 0.18), and moderate support for a higher probability of day/night detection in the winter for coyote (𝜂 = 0.84) and bobcat (𝜂 = 0.70). For the herbivore/small mammals, gray, and red squirrels have a higher probability of detection in day/night in the summer (𝜂 < 0.90) while white‐tailed deer, eastern cottontail, and eastern chipmunk have a higher probability of detection in day/night during the winter season (𝜂 > 0.90; Figure 4). Raccoons were the only urban‐adapted omnivore that had a higher probability of detection in day/night during the winter season (𝜂 > 0.90; Figure 4). There were observed differences across seasons in the predicted posterior probability distributions for state detection probability for all species. This difference in probabilities between day, night, and day/night states across seasons indicates that there is heterogeneity in each species' detection probabilities across states (Appendices 5 and 6).

**FIGURE 5:**
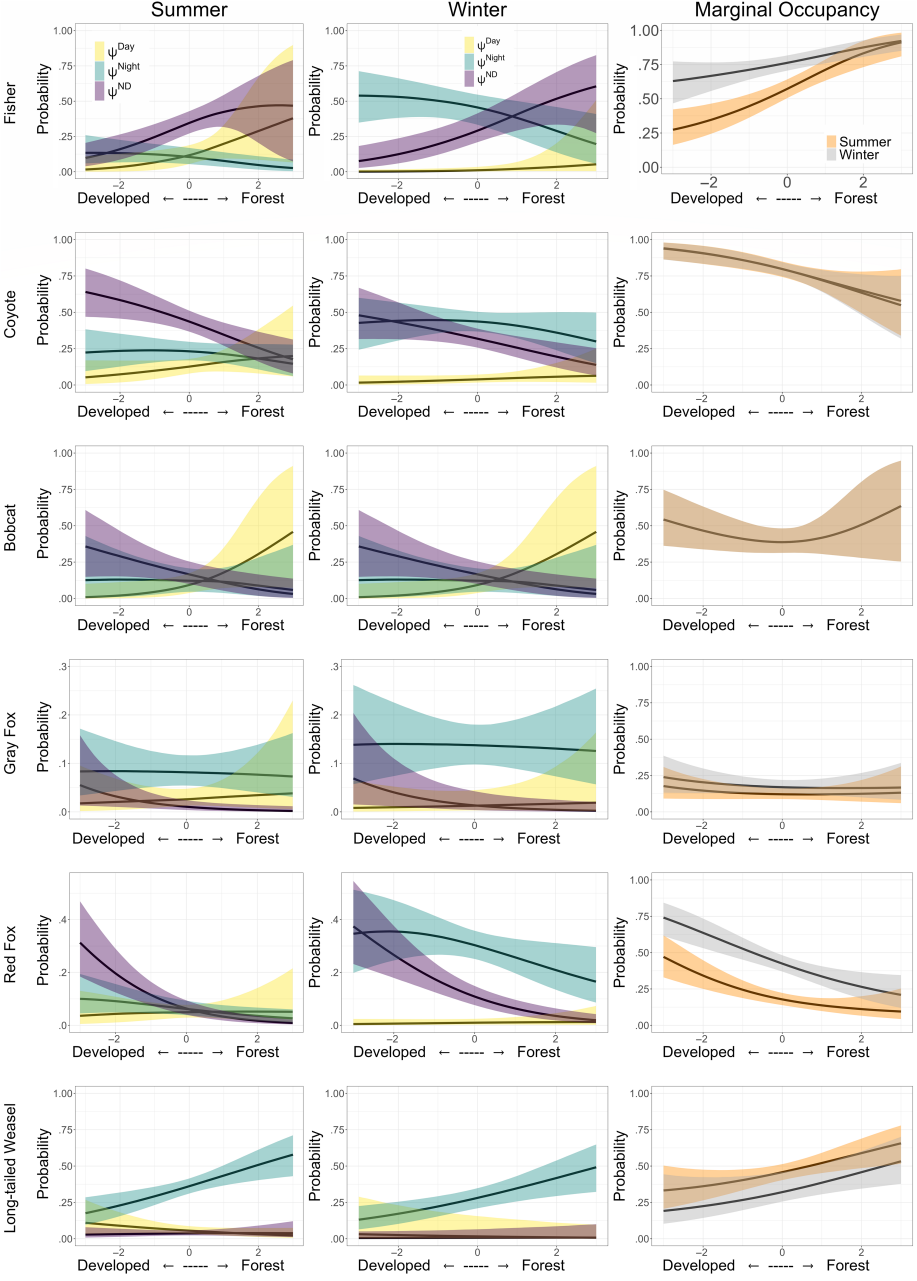
Predicted probability of seasonal and multistate diel occupancy and marginal occupancy (site occurrence, regardless of state; 𝝍^2^ + 𝝍^3^ + 𝝍^4^) for mesocarnivores. The solid black line represents the median and the colored envelopes indicate the 95% credible intervals. For each species, the predicted probability of site occupancy for each occupancy state (Day, Night, or Day/Night) is plotted for varying levels of *PCA* where positive values indicate more forest cover and negative values indicate more development. The most supported model for bobcat did not include a parameter for season, so no differences are shown between summer and winter.

**FIGURE 6:**
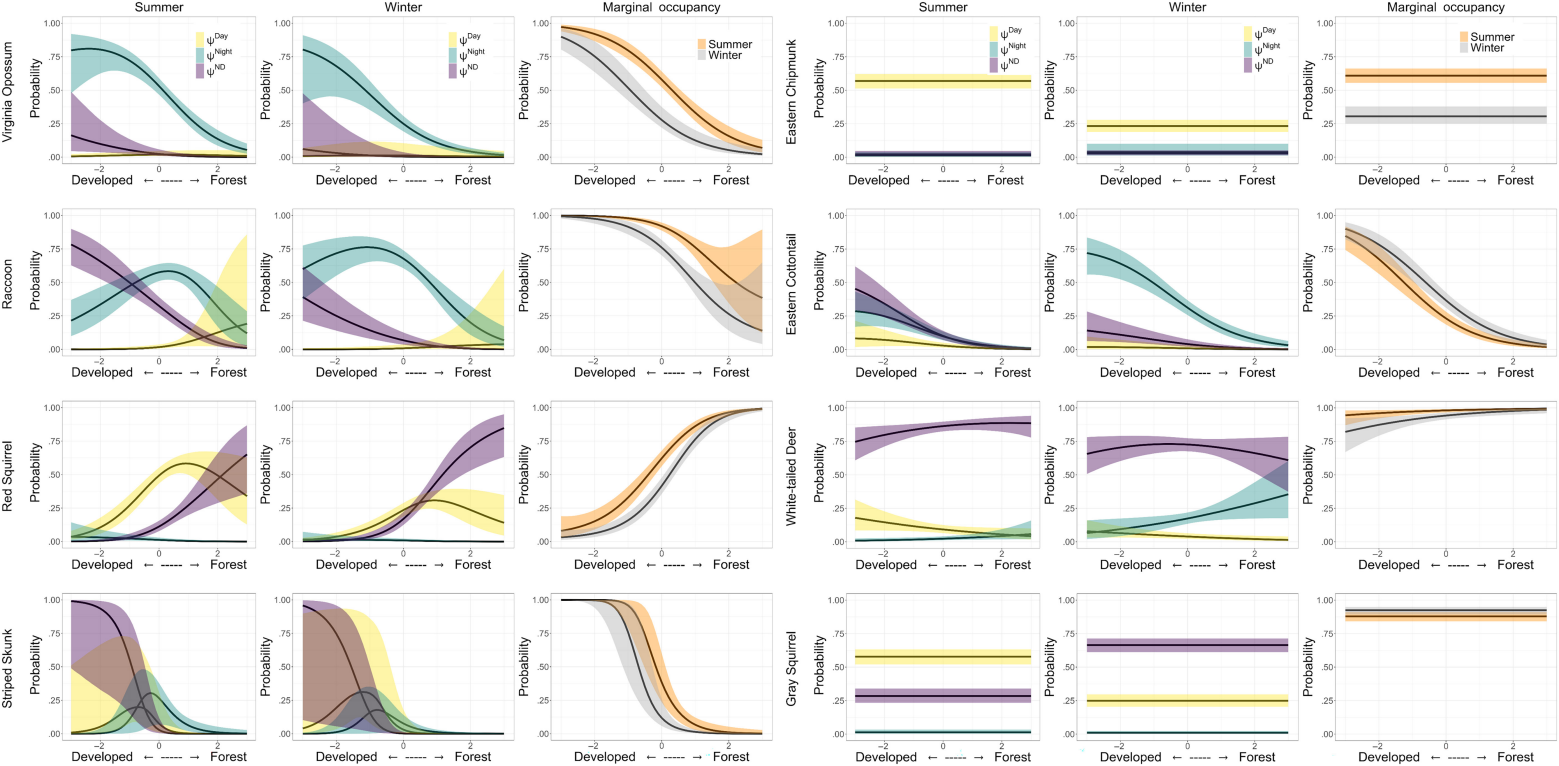
Predicted probability of seasonal and multistate diel occupancy and marginal occupancy (site occurrence, regardless of state; 𝝍^2^ + 𝝍^3^ + 𝝍^4^) for urban‐adapted omnivores and herbivores/small mammals. The solid black line represents the median and the colored envelopes indicate the 95% credible intervals. For each species, the predicted probability of site occupancy for each occupancy state (Day, Night, or Day/Night) is plotted for varying levels of *PCA* where positive values indicate more forest cover and negative values indicate more development. The most supported model for bobcat did not include a parameter for season, so no differences are shown between summer and winter.

We used the models for each species to predict seasonal occupancy given variation in the amount of development or forest cover at a site. All mesocarnivores have an increasing probability of using a site during the day as the forest cover increases (Figure 5). Fisher, coyote, and red fox have higher probability of using a site at night if there is more development, and long‐tailed weasels have a higher probability of using a site at night if the site has more forest cover. There were clear seasonal differences in predicted state occupancy in fisher, as they have a higher probability of using a site at night in the winter compared to the summer season. Coyote occupancy is high in both seasons at sites with more development, however they are more likely to use a site during the day if there is more forest cover. The marginal occupancy probability, or site occupancy regardless of diel state, increased with increasing forest cover during both winter and summer for fishers and long‐tailed weasels, and decreased for coyotes and red fox. There was more variation in predicted occupancy for the herbivore/small mammal and urban‐adapted omnivore species (Figure 6). Predicted state occupancy in summer for raccoons at sites with more development was highest in the day/night‐use state, and lowest in the day‐use state, and in the winter the predicted occupancy at sites with more development was highest in the night‐use state. All three urban‐adapted omnivores had a higher probability of marginal occupancy in sites with increasing development, regardless of season. Eastern cottontail was the only herbivore/small mammals that had a higher probability of marginal occupancy in sites with increasing development, while the opposite is true for white‐tailed deer and red squirrel. While the marginal occupancy probability trends were the same regardless of season for all species, there is clear heterogeneity in the predicted seasonal diel site occupancy.

## 4 DISCUSSION

In an increasingly human‐altered landscape, understanding wildlife habitats is crucial for conservation efforts. By studying the relationship between spatial habitat use and variation in use within a diel period, species occurrence can be modeled and predicted more accurately and effectively (Rivera et al., 2022). While previous studies have examined the diel activity of species across varying degrees of human‐developed landscapes based on camera trap detections, we took a multi‐state modeling approach to determine not only where species are likely to occur, but when they are likely to occur at these sites on the landscape. We found that site occupancy varied by season, diel period, and extent of forest, or development for many common mammal species, and that the probability of detecting all species in diel time varied by climatic season. By approaching the occupancy analysis with both spatial and temporal considerations we can make better predictions about how a mammal community can adapt to a landscape that is affected by disturbance and loss of spatial habitat, temporal habitat, or both.

We found the six mesocarnivore species observed in our study area to vary in their response to anthropogenic development (i.e., roads, housing). Recent studies have found that occupancy of some mesocarnivore species including bobcat and coyote is positively influenced by increasing distance from developed areas (Cove et al., 2019). As such, we hypothesized that mesocarnivores would have higher occupancy in sites with higher levels of anthropogenic disturbance only during the night‐use state, and that increasing anthropogenic disturbance would generally negatively affect occupancy in this species group. Our hypotheses were partially supported and we found that some species were more sensitive to the extent of development (i.e., fisher, bobcat, and long‐tailed weasel) and either had lower probabilities of occupying sites with higher development, or made adjustments to the diel time in which they occupied these sites, while other species were less sensitive and occupied areas with higher levels of development (i.e., coyote and red fox) by maintaining more nocturnal habits. This result agrees with Lesmeister et al. (2015) who found that coyotes, red fox, and gray fox were more likely to be nocturnal than diurnal or crepuscular. Bobcats, fishers, and long‐tailed weasels are not generally confined to nocturnal or diurnal habits (Chamberlain et al., 1998; Powell, 1993) and are detected on camera traps throughout the diel period. We found, however, that diel occupancy of a site (during the day only, during night only, or during both day and night) varied by the extent of forest cover and development at the site. Bobcats were only likely to occupy sites during the day if the site had a greater amount of forest cover, but the probability of using a site during the day/night‐use state was higher at sites with more development. Long‐tailed weasels were more likely to occupy sites at night than in the other diel states, but this was also driven by an increasing amount of forest cover. In addition to spatial and diel variation in occupancy, fisher also displayed variation in seasonal occupancy and were more likely to occupy sites with a greater amount of development at night during the winter than in the summer. The variation we observed in these species indicates that both season and diel period can lead to sites with increased amounts of development to be unavailable to these species at certain periods of the day (i.e., temporal habitat loss).

In contrast to these mesocarnivore species that had a strong response to development in their diel occupancy, both red fox and coyote were able to exploit areas of increasing development and had a higher probability of occupying these sites during the day/night‐use state and during the night compared to sites with increased forest cover. This distinction between groups of mesocarnivores is generally in‐line with prior research showing that coyotes are able to exploit more urbanized locations by constraining activity to night time when there is less risk from humans and traffic (Gallo et al., 2022; Riley et al., 2003; Tigas et al., 2002) while bobcats avoid developed areas if natural spaces are available (Parsons et al., 2019; Riley, 2006; Riley et al., 2003; Rodriguez et al., 2021), and fishers shift peaks in activity in response to risks such as increased traffic (LaPoint, 2013). Our analyses refine these previously known trends by incorporating the diel time of occupancy with extent of development and forest cover at sites that are occupied.

The urban‐adapted omnivores (raccoon, striped skunk, Virginia opossum) are typically classified as nocturnal animals, and thus we did not expect to see high occurrence during the day state regardless of the landscape characteristic or season and hypothesized that they would occupy areas with increased development primarily at night. These species have been shown to benefit from occupying landscapes with a relatively large anthropogenic footprint (Prange & Gehrt, 2004; Rodriguez et al., 2021). This may be due to increased availability of novel food sources and den sites, or the predation protection and limits on competition as a result of other larger species avoiding these more developed areas (Wang et al., 2015). While occupancy of a site during the day is minimal for all three species, there was observed variation between occupancy solely at night and occupancy of the day/night state both across seasons and with varying amounts of development (Figure 6). The overall low probability of occupancy in areas with increasing forest cover indicates that these species are well adapted to exploiting more developed areas, but because they are generally using these sites at night when other mesocarnivores are using these locations, it is not likely that these locations are used as a way of avoiding other species, and more likely because of the increased resources available at these sites.

Red squirrels are forest obligate species (Steele, 1998) and were the only species of the herbivore/small mammal group that had a strong occupancy response to the extent of forest cover. They are a typically diurnal species, and as expected, occupancy probability was highest in forests during the day‐use or day/night‐use state. Eastern cottontail typically occupy shrub habitats but are also associated with suburban landscapes. However, we did not include a landscape covariate for shrub habitats specifically, so it is unclear whether the predicted higher occupancy in all diel states at sites with a higher level of development is a result of the species avoidance for more open‐forested areas, (see O'Connor & Rittenhouse, 2017), or a true preference for occupying developed areas (see Jones et al., 2016). White‐tailed deer are a very common species both in natural forested areas and residential areas (Kilpatrick & Spohr, 2000) within our study area. As expected, they have an overall high probability of occupancy in the day/night‐use state with little variation between sites with development or forests. Simply, white‐tailed deer are active throughout the day and night on most conserved lands in Rhode Island. There was, however, a seasonal shift in increased night occupancy in forested areas during the winter season. This may be a result of increased nocturnality during the harvest season in areas that permit hunting (Kilgo et al., 1998).

Seasonality was a driver for variation in diel site occupancy for all mammal species but bobcat, and a driver for variation in diel detection probability for all species. Due to differences in the availability (total number of hours) of day and night between seasons, increases in night occupancy during the winter could be a result of increased availability or behavioral shifts. Additionally, many species exhibit variation in diel activity between summer and winter as a result of life history traits such as breeding and young‐rearing (Eriksen et al., 2011; Martin et al., 2010) which may affect when and where a species occupies a landscape. Bimodal activity, or crepuscular activity can also occur seasonally. Although this activity pattern is not explicitly modeled as an occupancy state in this MSDOM framework, it is captured under the day/night‐use state which allows for a more streamlined modeling process. While none of the mesocarnivores or urban‐adapted mammals had significant effects for seasonal differences across the day/night‐use state, the predicted day/night occupancy trended higher in the summer for fisher, coyote, red fox, and racoons compared to the winter season, and this may be a result of either increased daytime availability or site fidelity due to young‐rearing or constricted home range. Additionally, comparing the results of the predictive modeling for the mesocarnivores with some of their common prey species, there is not a clear distinction in the temporal and spatial site use. Prior studies have found that the presence of coyotes can have negative effects on the occupancy prey species including white‐tailed deer, squirrels (*Sciurus* spp.), and eastern cottontails (Jones et al., 2016), as well as other carnivore species, including weasels (*Mustela* spp.; Gompper et al., 2016). Although we did not test for species‐specific interactions, and acknowledge that predator avoidance is likely occurring, in these models the variation in diel occupancy does not appear to be driven by the presence/absence of predators at a spatial location in diel time. More likely, the occupancy of a site is driven by seasonality and the extent of human activity around a site.

It is well known that there is heterogeneity in where species occur on the landscape, and accounting for that heterogeneity in occupancy and detection probability (MacKenzie et al., 2017) improves the understanding of where species are likely to occur. There has also been recent interest in understanding variation in diel activity as it relates to anthropogenic disturbance (Gaston, 2019; Gaynor et al., 2018). Our findings show in a multi‐state diel occupancy modeling framework, there is heterogeneity both when and where members of a mammal community occupy a landscape, and in our ability to detect species during different states. Combining spatial and diel variation and accounting for this heterogeneity allows for more accurate predictions and reduced bias in identifying the habitats and resources that are important to a species in both space and time (Rivera et al., 2022). Understanding spatial and temporal variation will become increasingly important for effective conservation as species face loss of temporal habitat on a seasonal scale due to climatic shifts, and loss of diel habitat as anthropogenic pressures expand.

Anthropogenic development is increasing in North America (Alig et al., 2004; Hansen et al., 2005). In a region that already has a high anthropogenic footprint that may increase further, protection of remaining natural spaces is critically important for conservation. In Rhode Island, approximately 68% of forest land is in private ownership (Butler, 2018), and thus at potential risk for development. Our findings show that some mesocarnivore species are especially vulnerable to increased anthropogenic development, and loss of natural habitat may lead to a loss spatial habitat for long‐tailed weasels, temporal habitat in bobcats, or both for fishers and gray fox (Figure 5). As such, identifying and conserving natural spaces for wildlife is important to limit both spatial and temporal habitat loss, and using the diel activity and occupancy predictions will allow for a more targeted approach to land conservation and management.

## AUTHOR CONTRIBUTIONS

**Amy E. Mayer:** Conceptualization (supporting); data curation (equal); formal analysis (supporting); investigation (equal); methodology (equal); visualization (equal); writing – original draft (lead). **Laken S. Ganoe:** Data curation (equal); investigation (equal); methodology (equal); writing – review and editing (equal). **Charles Brown:** Funding acquisition (supporting); methodology (equal); supervision (supporting); writing – review and editing (equal). **Brian D. Gerber:** Conceptualization (lead); formal analysis (lead); funding acquisition (lead); methodology (equal); supervision (lead); visualization (equal); writing – original draft (supporting); writing – review and editing (equal).

## CONFLICT OF INTEREST STATEMENT

There are no conflicts of interest to declare.

## Data Availability

The data (multi‐state detection data for each species and covariates) and R code used to fit the models are archived and available on Zenodo: https://doi.org/10.5281/zenodo.8240191.

## APPENDIX 1

### 1.1

Radial distribution of independent detections plotted as a proportion of total detections for (A) mesocarnivore species and (B) herbivore/small mammal and urban‐adapted omnivore species in each season, summer and winter. The average sunrise and sunset times for the summer season were 05:39 and 20:00, respectively (14.36 h in the day period) and the average sunrise and sunset times for the winter season were 06:54 to 16:57, respectively (10.04 h in the day period).

## APPENDIX 2

### 2.1

Principal components analysis plot demonstrating the proportional variance explained by component 1 (85.1%) and the directionality of support for forest cover (+) and all anthropogenic development variables (road density, residential area, and housing unit index) (−).

## APPENDIX 3

### 3.1

Multi‐state diel occupancy modeling results by species. The top two most supported models for each species are reported with the corresponding conditional predictive ordinate (CPO) values. For each species, the model with the lowest CPO was selected to make inference on parameters. Full and reduced refer to the parameterization type of the model defining the day/night state (state 4). The variables in the parentheses indicate those specified to affect state occupancy (𝝍) and state detection probability (*p*).

**Table:**

Species	Top 2 models	CPO values
Bobcat	Full: 𝝍 (PCA), *p* (Season)	1085.648
	Full: 𝝍 (.), *p* (Season)	1087.761
Coyote	Full: 𝝍 (Season + PCA), *p* (Season)	6031.324
	Full: 𝝍 (Season), *p* (Season)	6037.405
Fisher	Full: 𝝍 (Season + PCA), *p* (Season)	4237.551
	Full: 𝝍 (Season + Prey), *p* (Season)	4242.072
Gray fox	Full: 𝝍 (Season + PCA), *p* (Season)	1074.748
	Full: 𝝍 (Season), *p* (Season)	1077.284
Red fox	Full: 𝝍 (Season + PCA), *p* (Season)	2673.846
	Full: 𝝍 (Season + Prey), *p* (Season)	2683.274
Long‐tailed weasel	Reduced: 𝝍 (Season + PCA), *p* (Season)	2343.568
	Reduced: 𝝍 (Season + PCA), *p* (Season)	2348.080
White‐tailed deer	Full: 𝝍 (Season + PCA), *p* (Season)	17482.04
	Full: 𝝍 (Season), *p* (Season)	17484.73
Eastern cottontail	Full: 𝝍 (Season + PCA), *p* (Season)	3950.644
	Reduced: 𝝍 (Season + PCA), *p* (Season)	3969.540
Gray squirrel	Full: 𝝍 (Season), *p* (Season)	18986.46
	Full: 𝝍 (Season + PCA), *p* (Season)	18990.46
Red squirrel	Full: 𝝍 (Season + PCA), *p* (Season)	7679.950
	Full: 𝝍 (PCA), *p* (Season)	7698.528
Eastern chipmunk	Full: 𝝍 (Season), *p* (Season)	5525.677
	Reduced: 𝝍 (Season + PCA), *p* (Season)	5531.390
Raccoon	Full: 𝝍 (Season + PCA), *p* (Season)	8074.340
	Full: 𝝍 (Season), *p* (Season)	8108.570
Striped skunk	Reduced: 𝝍 (Season + PCA), *p* (Season)	886.369
	Reduced: 𝝍 (PCA), *p* (Season)	893.468
Virginia opossum	Reduced: 𝝍 (Season + PCA), *p* (Season)	3529.462
	Full: 𝝍 (Season + PCA), *p* (Season)	3545.323

## APPENDIX 4

### 4.1

Summary of interpretation of multi‐state diel occupancy modeling results for mesocarnivores (red cells), urban‐adapted omnivores (yellow cells), and herbivore/small mammals (green cells). If a species is listed, this indicates that the covariate (PCA: extent of forest cover or development; season) significantly affected diel site occupancy for that species.

**Table:**

	Positive effect on site diel occupancy given:											
	Increasing forest			Increasing development			Summer			Winter		
Day state	Fisher, bobcat				Skunk, opossum	Cottontail, red squirrel	Fisher, coyote, red fox	Raccoon	Deer, chipmunk			Red squirrel
Night state	Weasel		Deer	Coyote, red fox	Raccoon, skunk, opossum	Cottontail	Weasel	Raccoon, opossum, skunk		Fisher, coyote, gray fox, red fox		Cottontail, deer
Day/Night state			Cottontail, red squirrel	Bobcat, gray fox		Deer						Gray squirrel, red squirrel

## APPENDIX 5

### 5.1

Predicted posterior probability distributions of state detection probability for mesocarnivores by season. P^ND2^ is the probability of detection in the day given the species occupies the day/night state, P^ND3^ is the probability of detection at night given the species occupies the day/night state, and P^ND4^ is the probability of detection at night and day given the species occupies the day/night state. Silhouettes embedded using R package rphylopic (Gearty & Jones, 2023).

## APPENDIX 6

### 6.1

Predicted posterior probability distributions of state detection probability for herbivore/small mammals and urban‐adapted omnivores by season. P^ND2^ is the probability of detection in the day given the species occupies the day/night state, P^ND3^ is the probability of detection at night given the species occupies the day/night state, and P^ND4^ is the probability of detection at night and day given the species occupies the day/night state. Silhouettes embedded using R package rphylopic (Gearty & Jones, 2023).
